# Nasolabial Cyst: a Diagnostic Dilemma

**DOI:** 10.30476/DENTJODS.2021.87394.1260

**Published:** 2022-03

**Authors:** Surej Kumar LK, Dilna Sidharthan, Benoy Stanly, Cucoo Mariam Mathew

**Affiliations:** 1 Dept. of Oral and Maxillofacial surgery, KIMS Hospital, Anayara, Trivandrum, India; 2 Maxillofacial Surgeon, Former Trainee, KIMS Hospital, Anayara, Trivandrum, India; 3 KIMS Dental & International Patients, KIMS Hospital, Anayara, Trivandrum, India

**Keywords:** Nasolabial, Cyst, Klestadt’s cyst, Nonodontogenic cyst

## Abstract

Nasolabial cyst or klestadt’s cyst is a rare nonodontogenic lesion occurring in the maxillofacial region. It is commonly seen in the nasoalveolar area, lateral to ala of nose. It is usually asymptomatic and is often ignored by the patient unless it enlarges in size resulting in cosmetic deformities. It is often challenging not only to maxillofacial surgeons but also to other head and neck surgeons. It is often misdiagnosed as other common dental related or odontogenic lesions and mistreated. Here we are describing such a surgically managed case as a case report and also discussing the etiopathogenesis and management.

## Introduction

Nasolabial cyst is a benign slow growing extra osseous lesion usually seen in the nasal alar region which was first reported by Emil Zukerkandl [ 1 ]. Klestadt [ 2 ] had studied about the same in the year 1953 and the lesion was known as Klestadt cyst. The incidence of nasolabial cyst is found to be 0.7% among all maxillofacial cysts [ 3 ]. It is common in fourth to fifth decades of life [ 4 ]. Often these lesions have gone unnoticed or misdiagnosed, as they are not evident on common radiographs. Patient may report with clinical symptoms related to cosmetic deformity and large lesions may result in nasal obstruction. Diagnosis is usually made by clinical findings and with other imaging modalities. 

## Case Presentation

A 48-year-old female reported to the Department of Oral and Maxillofacial surgery (OMFS) with a painless swelling on the left side of the nose, which was noticed 8 months back. No history of trauma was elicited. Swelling was persistent and was enlarging in size, as per history.

**Figure 1:**
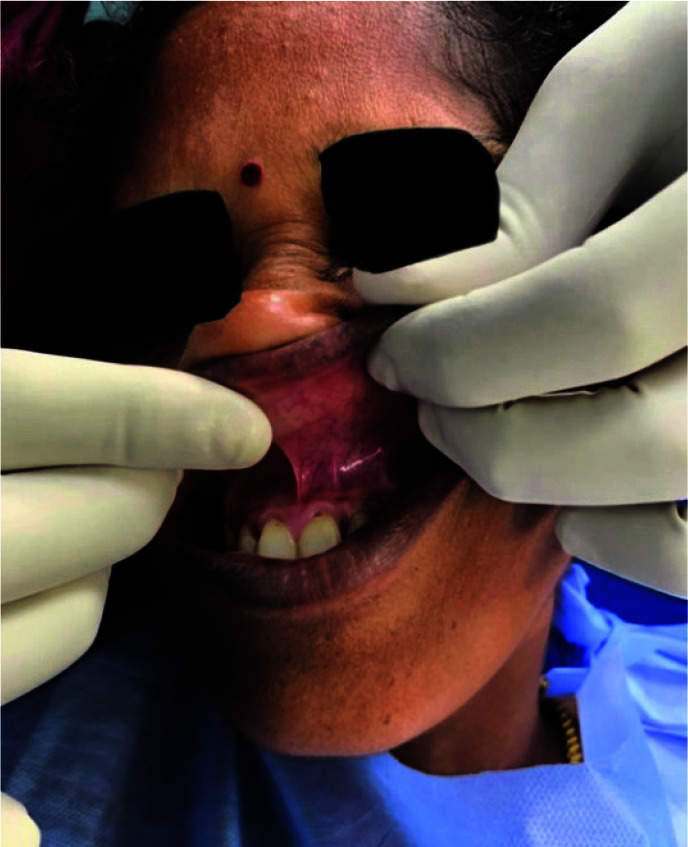
Intraoral view of lesion

On extraoral examination, a swelling sized 2×2 cm with ill-defined borders was elicited, lateral to the ala of the nose. The surface was smooth, and no punctum was seen. The swelling was soft and cystic with mild tenderness on palpation. The swelling was palpable intra-orally into the buccal vestibule of the upper left canine region (Figure 1). 

It was nonmobile and cystic in nature. Infective foci of dental origin were ruled out, with thermal and electrical vitality testing of teeth, Hence the possibility of a radicular cyst or dentoalveolar abscess was ruled out.

**Figure 2:**
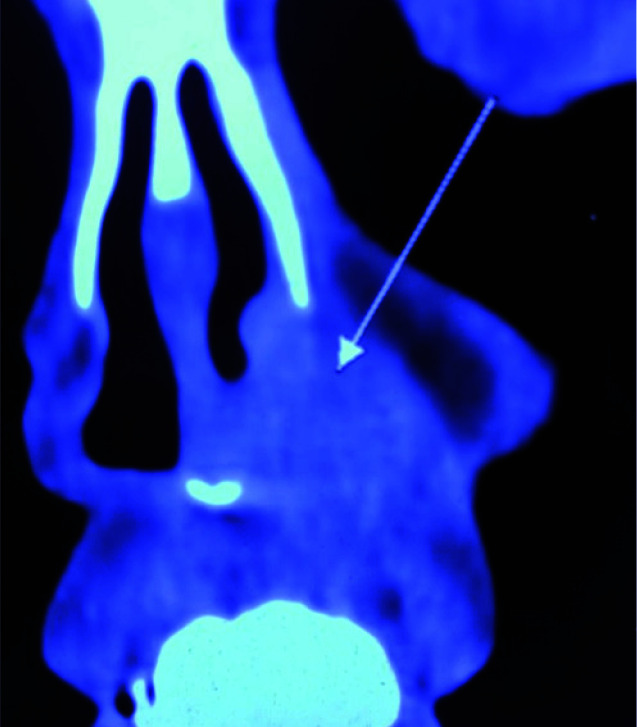
CT coronal view

**Figure 3:**
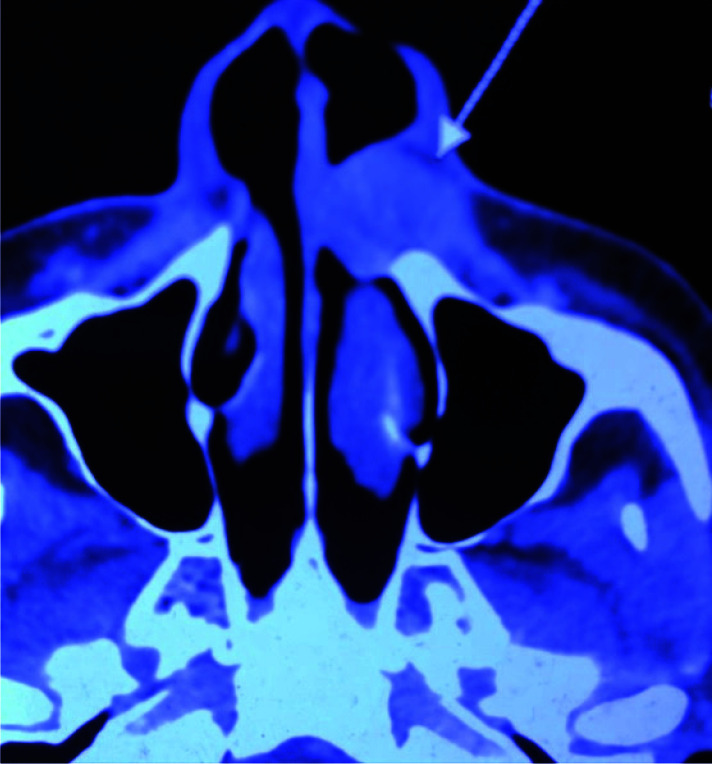
CT axial view

A computerized tomographic (CT) scan revealed a well-defined round soft tissue lesion in the lateral pyriform aperture (Figures 2 and 3). 

Based on the findings, a provisional diagnosis of a non-odontogenic/ soft tissue cyst or tumor was made, which included nasolabial cyst, lipoma, dermoid cyst and epidermoid cyst. An excision biopsy was planned under local anesthesia.

**Figure 4:**
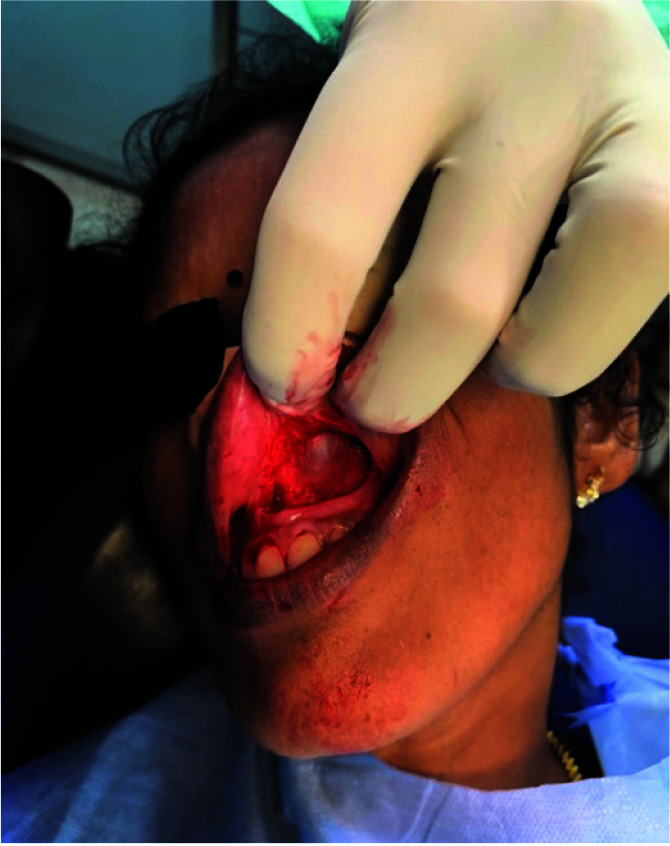
After incision

Left infraorbital nerve block and local infiltration in vestibule and palatally infiltration was performed for the patient. Then, intraorally vestibular incision at level of nasal floor only through the mucosa, and careful blunt dissection done to deepen to the lesion (Figure 4) was performed.

**Figure 5:**
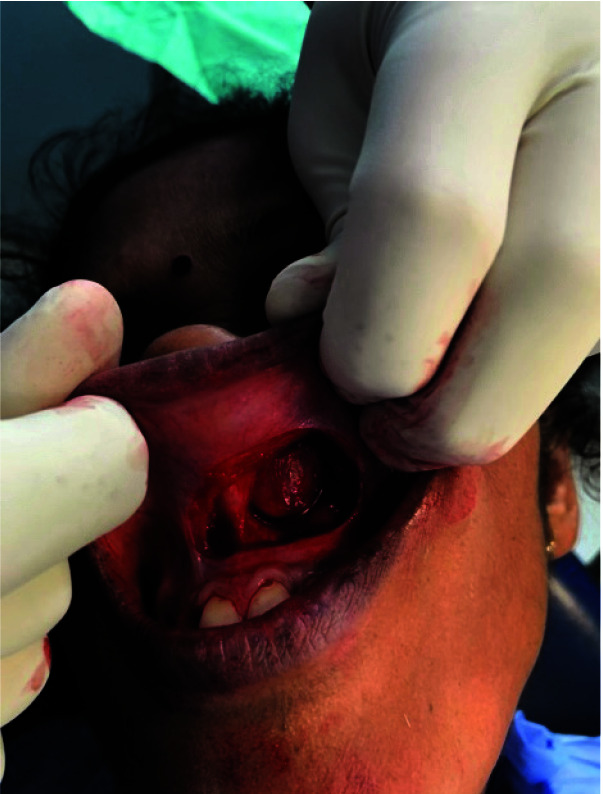
Exposed lesion with capsule

**Figure 6:**
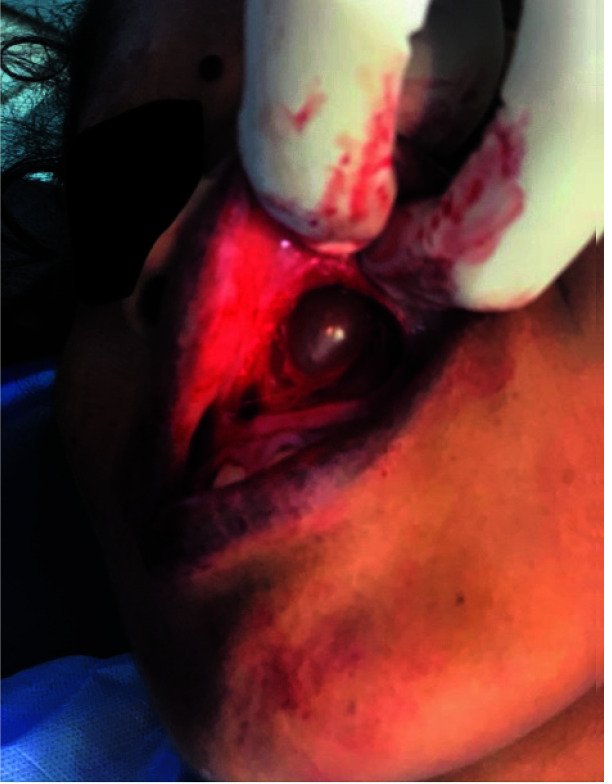
Capsule opened to expose the lesion

**Figure 7:**
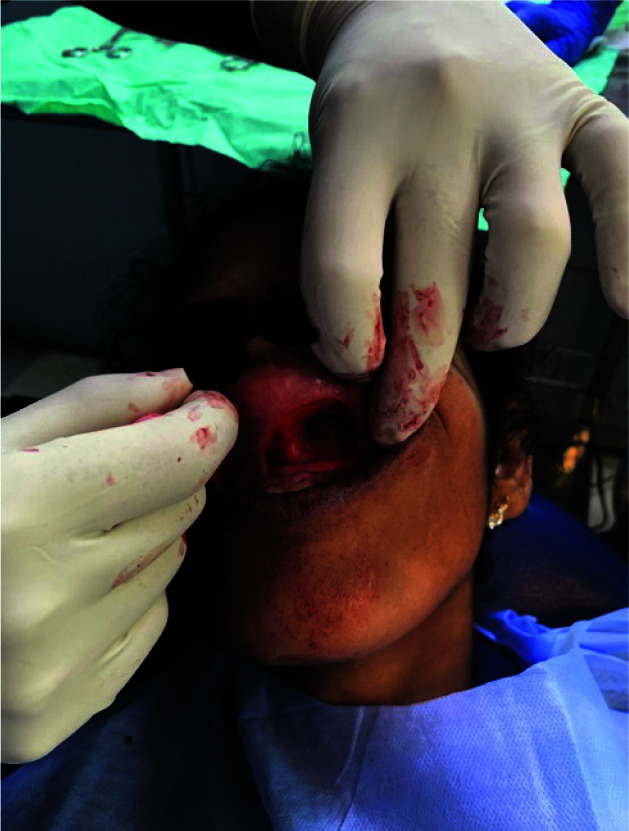
After excision showing the defect area

**Figure 8:**
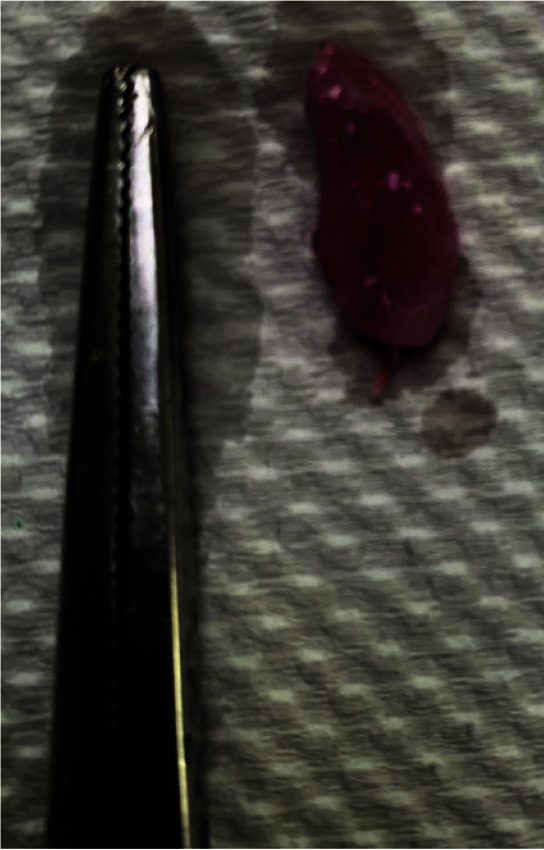
Excised specimen

**Figure 9:**
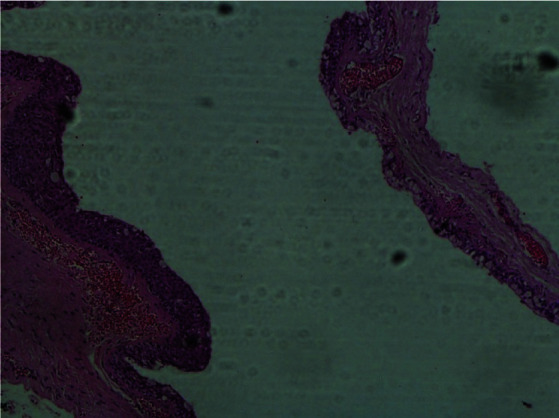
Histopathologic specimen

Once the lesion was exposed, blunt dissection was proceeded around to free it from the floor of nose (Figure 5). Intraoperative inspection revealed that the lesion was surrounded by a thick lining, which was opened to expose the cystic lesion (Figure 6), surrounded the lesion. Once the lesion was freed from the nasal floor, posterior dissection was done to free the lesion completely and removed in total (Figure 7). Hemostasis was obtained and the wound was closed with 3/0 vicryl. The excised specimen (Figure 8) was sent for histopathological examination. Post operative period was uneventful. Histopathological examination showed cyst lined by double layered goblet cell rich columnar epithelium with focal squamous metaplasia (Figure 9) and the diagnosis was consistent with nasolabial cyst. 

## Discussion

Nasolabial cyst is purely a soft tissue nonodontogenic cyst though it has been classified under jaw cysts. It is also termed as the mucoid cyst of nose, nasal cyst and sometimes even as nasoalveolar cyst. Most of the reported cases have been unilateral in nature, but bilateral cases have also been reported in the literatures [ 5 - 7 ]. The etiopathogenesis is unclear but three kinds of theories have been proposed for the formation of nasolabial cyst [ 6 , 8 ]. These include (1) embryological detention of cells in the medial and lateral nasal wall, (2) detention of cell in the nasolacrimal channel and (3) possible trauma accelerating its formation.

Nasolabial cyst exhibits wide variety of clinical symptoms and usually varies from a painless swelling to an area of localised numbness. Usually they are found near the opening of the nasolacrimal duct in the inferior meatus. A soft oval palpable mass is usually seen in ala area. Bimanual palpation of nasal floor and labial sulcus often confirms its position. Secondary infection may result in tenderness and pus accumulation followed by spontaneous rupture and drainage into oral or nasal cavity. Rarely, it causes difficulty in breathing or nasal blockage unless greater dimensions are achieved to produce symptoms. Extraorally, the cyst may obliterate the nasolabial fold, often lifting the nasal ala causing deformity of the nostril. Similar scenario was also observed in our case. Rarely dacrocystitis may occur with epiphora [ 9 - 10 ] if the nasolacrimal opening is blocked, but our case was not associated with such a condition. Proper diagnosis has to be made as many pathological lesions mimic the same lesion. Primarily a dentoalveolar abscess has to be excluded and it can easily be done by simple radiographs and by vitality testing of teeth. Certain nonodontogenic intra osseous cyst like nasolabial palatine cyst may also mimic the same, but this can be excluded by extra osseous nature of nasolabial cyst and can be confirmed with imaging modalities like CT or magnetic resonance imaging (MRI) as in the presented case. History also plays an important role in diagnosis as we can rule out the childhood lesions like dermoid and epidermis cyst.

Diagnosis is usually made by clinical findings with CT or MRI. Several imaging modalities may be used. Since without bone involvement, it is rarely identified in plain radiographs. Though MRI gives good soft tissue definition, CT is preferable as it is less expensive. Ultra sound can also be a diagnostic method and has advantages over other invasive techniques [ 11 - 12 ]. 

Surgical excision is the treatment of choice with less morbidity. An intra oral approach is ideal with good cosmetic results. Other modalities include endoscopic excision or even marsupialization [ 13 - 14 ]. The Neumann [ 15 ] incision is found to be useful for complete cyst excision and best access to the pyriform aperture. 

Intra operative nasal mucosal perforation is a common complication which can be sutured or small perforations will heal spontaneously even if left untreated. Such complications were not observed in our case. Recurrence is usually rare on complete removal of lining and malignant transformation has not been reported but cellular changes have been reported in some cases in literature reviews. Informed consent from patient obtained.

## Conclusion

Nasolabial cysts are rare soft tissue cysts less commonly seen in the population. The differential diagnosis of normal anatomical variation in the area and other vascular lesions should be kept in mind before surgical management. It is usually confirmed by clinical examinations aided with common imaging modalities. Surgical excision gives excellent results with less morbidity and recurrence.

## Conflict of Interest

The authors declare that they have no conflict of interest.
